# Mechanism-based therapeutic strategies in cardiovascular-kidney-metabolic syndrome: integrating biological pathways with evidence from clinical trials

**DOI:** 10.3389/fendo.2026.1987811

**Published:** 2026-10-22

**Authors:** John Nzobokela, Simweene C. Choolwe, Sepiso K. Masenga

**Affiliations:** 1 Department of Cardiovascular Science and Metabolic Diseases, Livingstone Center for Prevention and Translational Science, Livingstone, Zambia; 2 Department of Pathology and Microbiology, School of Medicine and Health Sciences, Mulungushi University, Livingstone, Zambia; 3 Department of Medicine, Vanderbilt University Medical Center, Nashville, TN, United States

**Keywords:** cardiovascular-kidney-metabolic syndrome, combination therapy, GLP-1 receptor agonists, mechanism-based therapy, SGLT2 inhibitors

## Abstract

Cardiovascular-kidney-metabolic (CKM) syndrome describes the close relationship between metabolic dysfunction, chronic kidney disease, and cardiovascular disease, which share several biological pathways and frequently progress together. This review examines the mechanisms and clinical trial evidence supporting established and emerging therapies relevant to CKM syndrome. Sodium-glucose cotransporter 2 inhibitors, glucagon-like peptide-1 receptor agonists, renin-angiotensin system inhibitors, mineralocorticoid receptor antagonists, and lipid-lowering therapies have shown cardiovascular, kidney, and metabolic benefits across several high-risk populations. However, most evidence comes from trials enrolling patients with individual conditions, such as type 2 diabetes, chronic kidney disease, heart failure, obesity, or atherosclerotic cardiovascular disease, rather than populations defined by CKM stage or phenotype. Evidence for the added clinical benefit of specific treatment combinations also remains limited. Newer therapies targeting aldosterone synthesis, endothelin signaling, inflammation, incretin pathways, and lipid-related pathways may broaden treatment options. In clinical practice, CKM stage and the predominant clinical phenotype may help identify treatment priorities while accounting for coexisting conditions, safety, treatment burden, cost, and access. This approach may provide a practical basis for selecting therapies across the CKM spectrum, although prospective studies are needed to determine whether phenotype-directed treatment improves clinical outcomes.

## 1 Introduction

### 1.1 Defining CKM syndrome

Cardiovascular-kidney-metabolic (CKM) syndrome represents a conceptual and clinical advance beyond the traditional management of cardiovascular disease (CVD), chronic kidney disease (CKD), and metabolic dysfunction as separate, organ-specific conditions. The 2023 American Heart Association (AHA) Presidential Advisory formally defined CKM syndrome as the pathophysiological interrelationship among metabolic risk factors, CKD, and the cardiovascular system, driven by shared biological mechanisms that propagate bidirectional organ injury (1). This framework acknowledges that approximately 90% of adults in the United States have at least one CKM risk factor and that the rising global prevalence of obesity, type 2 diabetes (T2D), CKD, and CVD, particularly in low- and middle-income countries (LMICs), makes CKM syndrome an increasingly important public health challenge (2, 3).

The economic and humanistic burden of CKM syndrome is substantial. CKM-related conditions collectively contribute to considerable healthcare expenditures through high rates of cardiovascular events, kidney failure, heart failure (HF) hospitalization, and the cumulative costs of managing multimorbidity (4, 5). The epidemiological transition in LMICs, where the growing burden of metabolic disease intersects with constrained healthcare infrastructure, creates particular urgency for affordable, evidence-based, and equitable therapeutic strategies (6).

### 1.2 The AHA CKM staging framework

The AHA conceptualizes CKM syndrome as a five-stage continuum (Stages 0–4) integrating metabolic dysfunction, CKD, and CVD (**Figure 1**) (1):

**Figure 1:**
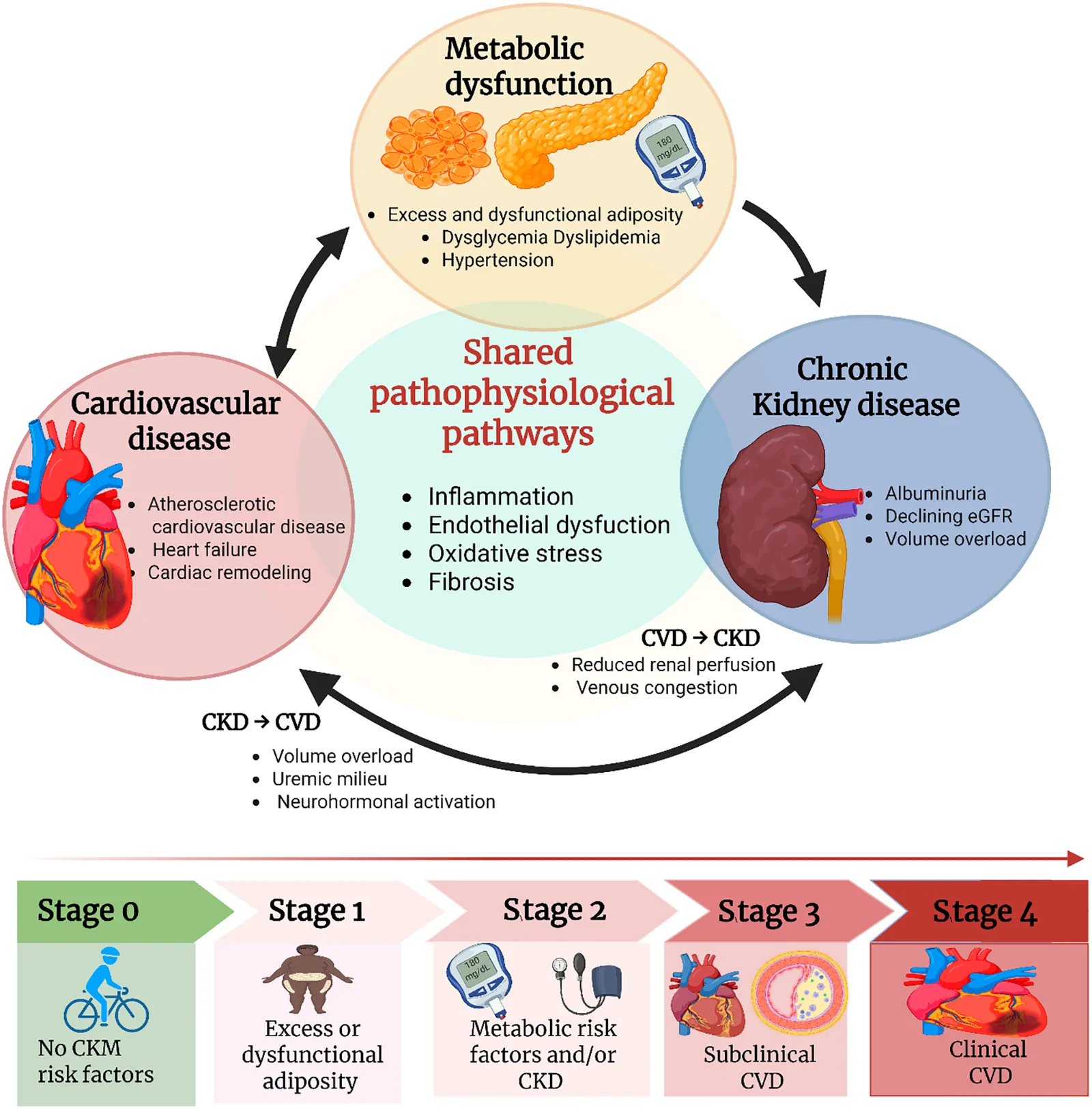
Interactions and progression across cardiovascular-kidney-metabolic syndrome. Metabolic dysfunction, cardiovascular disease, and chronic kidney disease are closely interconnected. Excess or dysfunctional adiposity, dysglycemia, dyslipidemia, and hypertension contribute to cardiovascular and kidney injury through inflammation, endothelial dysfunction, oxidative stress, and fibrosis. Cardiovascular disease can worsen kidney function through reduced renal perfusion and venous congestion, whereas kidney disease can aggravate cardiovascular disease through volume overload, the uremic milieu, and neurohormonal activation. These reciprocal effects can promote progressive multiorgan disease. CKM staging ranges from no CKM risk factors (Stage 0), to excess or dysfunctional adiposity (Stage 1), metabolic risk factors and/or CKD (Stage 2), subclinical cardiovascular disease (Stage 3), and clinical cardiovascular disease (Stage 4). CKM, cardiovascular-kidney-metabolic; CVD, cardiovascular disease; eGFR, estimated glomerular filtration rate. Created using BioRender, licensed under the BioRender Academic License.

- Stage 0: Preserved CKM health. Absence of CKM risk factors, with healthy adiposity, metabolic parameters, kidney function, and cardiovascular status.
- Stage 1: Excess or dysfunctional adiposity. Excess or dysfunctional adiposity without established metabolic risk factors or CKD, representing an early substrate for insulin resistance, inflammation, and subsequent CKM progression.
- Stage 2: Metabolic dysfunction and/or CKD. Presence of metabolic risk factors, including hypertension, hypertriglyceridemia, metabolic syndrome, or T2D, and/or moderate- to high-risk CKD, without clinical CVD.
- Stage 3: Subclinical CVD or high-risk equivalents. Evidence of subclinical CVD or conditions conferring comparably high cardiovascular risk, including very-high-risk CKD or high predicted CVD risk.
- Stage 4: Established CVD. Clinical CVD, including coronary heart disease, HF, stroke, peripheral artery disease, or atrial fibrillation, in the context of CKM risk factors. Stage 4a occurs without kidney failure, whereas Stage 4b includes concomitant kidney failure.

The 2026 AHA/ACC/ADA/ASN CKM Guideline further operationalizes this framework by linking CKM stage and cardiovascular risk to preventive and therapeutic decision-making (7), This staging continuum provides the clinical foundation for the mechanism-based therapeutic approach discussed in this review.

### 1.3 From siloed to integrated therapy

CKM-related conditions have traditionally been managed within organ-specific frameworks, with cardiovascular, kidney, and metabolic disorders treated largely as separate entities. Although effective for individual conditions, this fragmented approach may inadequately address the interconnected mechanisms that sustain residual cardiovascular and kidney risk (8, 9).

The emergence of therapies with benefits across organ systems, particularly SGLT2 inhibitors and GLP-1RAs, has accelerated a shift toward integrated CKM management. Clinical trials demonstrate cardiovascular and kidney benefits extending beyond their original glucose-lowering indications, supporting treatment strategies increasingly guided by shared biological pathways and overall CKM risk rather than isolated diagnoses (10, 11). However, uncertainty remains regarding therapeutic prioritization, optimal sequencing and combination, and the applicability of disease-specific trial evidence across the broader CKM spectrum.

### 1.4 Scope, methods, and organization

This narrative review evaluates mechanism-based therapeutic strategies for CKM syndrome. Relevant literature was identified through searches of PubMed/MEDLINE, Embase, and ClinicalTrials.gov, with a search cutoff of June 2026. Search terms included combinations of “cardiovascular-kidney-metabolic syndrome”, “CKM”, “SGLT2 inhibitors”, “GLP-1 receptor agonists”, “finerenone”, “mineralocorticoid receptor antagonist”, “RAAS inhibition”, “lipid-lowering”, “heart failure”, “chronic kidney disease”, “colchicine”, “aldosterone synthase inhibitor”, “endothelin receptor antagonist”, “inclisiran”, and “olpasiran”. Priority was given to randomized controlled trials (RCTs), major cardiovascular and kidney outcome trials, prespecified subgroup analyses, pooled meta-analyses, and relevant systematic reviews. The strength and consistency of the evidence were considered narratively, with attention to study design, population, effect estimates, and consistency across major trials.

## 2 Mechanistic targets in CKM syndrome

CKM syndrome arises from interacting metabolic, hemodynamic, inflammatory, and fibrotic pathways that link metabolic dysfunction with cardiovascular and kidney injury (**Figure 2**). Dysfunctional adiposity, insulin resistance, RAAS and mineralocorticoid receptor activation, altered tubular hemodynamics, atherogenic dyslipidemia, and chronic inflammation often coexist and reinforce organ damage. These pathways define the principal therapeutic targets considered in this review.

**Figure 2:**
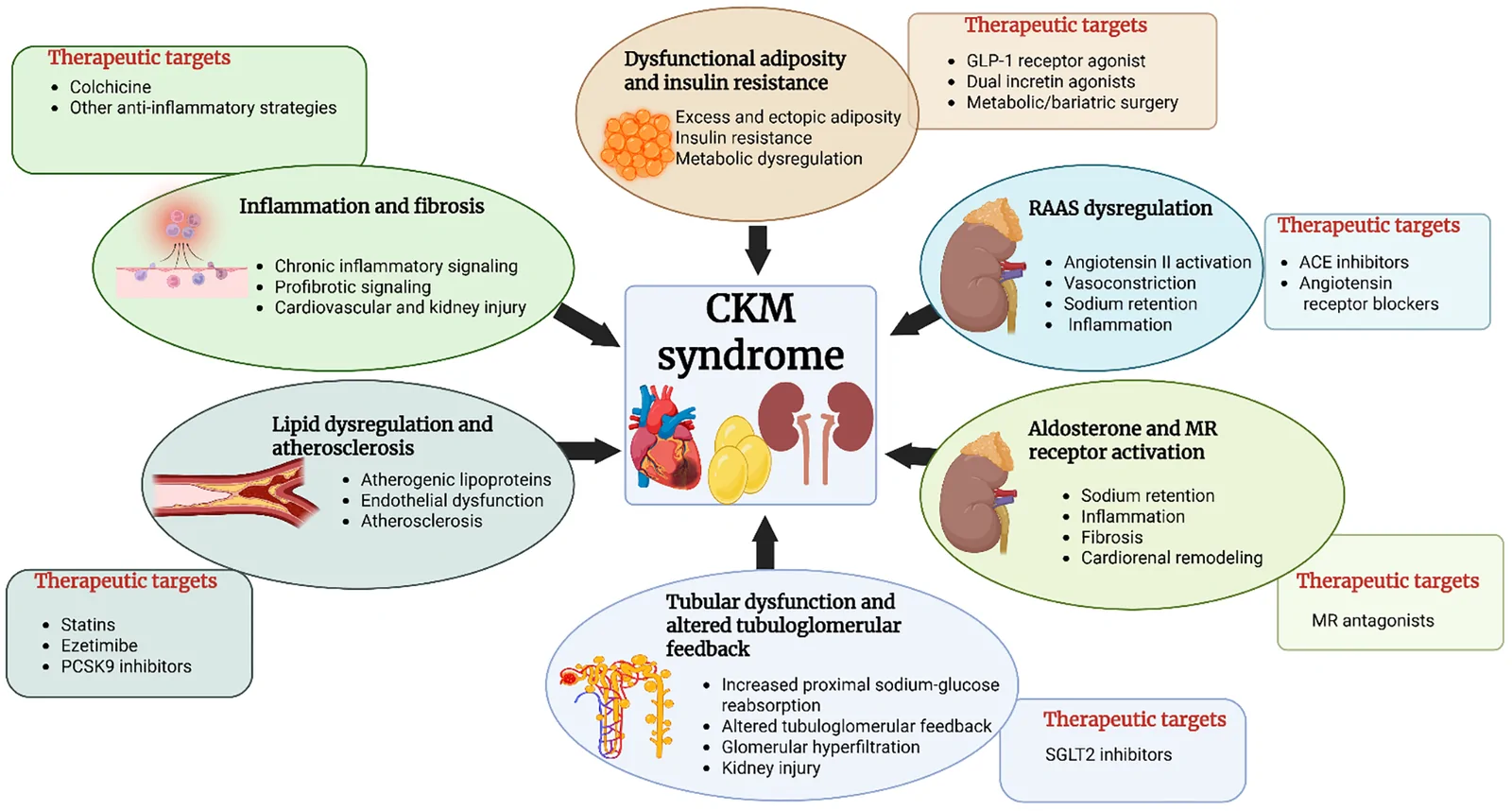
Mechanism-based therapeutic targeting across cardiovascular-kidney-metabolic syndrome. Cardiovascular-kidney-metabolic syndrome is driven by interconnected metabolic, hemodynamic, inflammatory, fibrotic, renal, and atherosclerotic pathways that provide complementary therapeutic targets. Dysfunctional adiposity and insulin resistance are targeted by GLP-1 receptor agonists, dual incretin agonists, and metabolic/bariatric surgery. RAAS dysregulation is targeted by ACE inhibitors and angiotensin receptor blockers, while mineralocorticoid receptor antagonists counter aldosterone-mediated sodium retention, inflammation, fibrosis, and cardiorenal remodeling. SGLT2 inhibitors reduce proximal sodium-glucose reabsorption and modulate tubuloglomerular feedback, addressing glomerular hyperfiltration and kidney injury. Statins, ezetimibe, and PCSK9 inhibitors target atherogenic lipid pathways, while colchicine and other anti-inflammatory strategies address components of persistent inflammatory signaling. Targeting these complementary pathways provides the mechanistic basis for integrated CKM therapy aimed at reducing cardiovascular, kidney, and metabolic risk. ACE, angiotensin-converting enzyme; CKM, cardiovascular-kidney-metabolic; GLP-1, glucagon-like peptide-1; MR, mineralocorticoid receptor; PCSK9, proprotein convertase subtilisin/kexin type 9; RAAS, renin-angiotensin-aldosterone system; SGLT2, sodium-glucose cotransporter 2. Created using BioRender, licensed under the BioRender Academic License.

### 2.1 Dysfunctional adiposity and insulin resistance

Visceral and ectopic adiposity promotes insulin resistance through increased circulating free fatty acids, ceramide accumulation, impaired insulin receptor signaling, and reduced adiponectin secretion (12, 13). The resulting metabolic disturbance is associated with hyperglycemia, hypertriglyceridemia, hypertension, and endothelial dysfunction. Ectopic fat accumulation in the liver, kidney, myocardium, and skeletal muscle further exposes these tissues to lipotoxicity and altered substrate utilization (14).

### 2.2 Chronic inflammation and fibrosis

Dysfunctional adipose tissue sustains low-grade inflammation through innate immune activation and increased signaling involving interleukin-1β (IL-1β), interleukin-6 (IL-6), tumor necrosis factor-α (TNF-α), and the NLRP3 inflammasome (15, 16). Macrophage infiltration and persistent inflammatory signaling are implicated in myocardial remodeling and diastolic dysfunction, tubulointerstitial injury, and atherosclerotic plaque formation (17). Transforming growth factor-β (TGF-β) drives extracellular matrix deposition and fibrosis in cardiac, kidney, and vascular tissues, linking persistent inflammation to structural organ damage (18). Galectin-3 and soluble suppression of tumorigenicity 2 (sST2) are associated with fibrotic activity and adverse cardiovascular and kidney outcomes.

### 2.3 RAAS dysregulation

Chronic renin-angiotensin-aldosterone system (RAAS) activation is a major hemodynamic and neurohormonal driver of cardiovascular and kidney injury (19). Reduced kidney perfusion, sympathetic activation, and metabolic dysfunction can sustain this response. Angiotensin II induces vasoconstriction, oxidative stress, inflammation, and fibrosis. Preferential efferent arteriolar constriction raises intraglomerular pressure, while myocardial signaling promotes cardiomyocyte hypertrophy and interstitial fibrosis. In the vasculature, angiotensin II impairs endothelial function and favors atherogenesis (20, 21).

### 2.4 Mineralocorticoid receptor activation

Aldosterone-mediated mineralocorticoid receptor (MR) activation extends the effects of RAAS dysregulation across the kidney, heart, and vasculature (22). In the kidney, MR activation increases sodium retention and drives tubular inflammation and interstitial fibrosis. In cardiovascular tissues, it favors endothelial dysfunction, vascular inflammation, myocardial fibrosis, and adverse remodeling (23). Adipose-derived signals may further influence aldosterone production. Cortisol can also activate the MR in tissues with limited 11β-hydroxysteroid dehydrogenase type 2 (11β-HSD2) activity, allowing MR signaling to occur independently of elevated circulating aldosterone (24).

### 2.5 Tubular dysfunction and altered tubuloglomerular feedback

Hyperglycemia increases sodium-glucose cotransporter 2 (SGLT2)-mediated glucose and sodium reabsorption in the proximal tubule, reducing sodium chloride delivery to the macula densa. The resulting alteration in tubuloglomerular feedback favors afferent arteriolar dilation, increased intraglomerular pressure, and glomerular hyperfiltration (25, 26). Increased proximal tubular workload is also associated with tubular hypertrophy and oxidative stress. These changes connect metabolic disturbance with progressive glomerular and tubular injury.

### 2.6 Lipid dysregulation and atherosclerosis

KM-associated dyslipidemia may include hypertriglyceridemia, reduced high-density lipoprotein cholesterol (HDL-C), increased small dense low-density lipoprotein (LDL) particles, and elevated lipoprotein(a) [Lp(a)] (27, 28). Insulin resistance increases hepatic very-low-density lipoprotein (VLDL) production and disrupts triglyceride-rich lipoprotein metabolism. LDL and Lp(a) facilitate lipid deposition within the arterial wall and plaque progression, while oxidized LDL promotes foam cell formation, endothelial dysfunction, and vascular inflammation (29).

### 2.7 Emerging pathways

Gut microbial metabolites may influence CKM biology. Dysbiosis can alter trimethylamine N-oxide (TMAO) and short-chain fatty acid production, with downstream effects on vascular inflammation, platelet activity, and metabolic homeostasis (30, 31). Epigenetic regulation and noncoding RNAs also influence metabolic, inflammatory, and fibrotic gene expression in adipocytes, cardiomyocytes, renal tubular cells, and vascular cells. Their clinical relevance as biomarkers or therapeutic targets in CKM syndrome remains under investigation (32).

## 3 Established therapeutic classes: mechanistic rationale and clinical evidence

### 3.1 Therapies targeting metabolic dysfunction and adiposity

Dysfunctional adiposity and insulin resistance are major drivers of CKM progression and therefore represent important therapeutic targets. Recent trials indicate that therapies targeting these pathways can improve cardiovascular, kidney, and metabolic outcomes, with benefits that extend beyond glycemic control alone.

#### 3.1.1 GLP-1 receptor agonists

Glucagon-like peptide-1 receptor agonists (GLP-1RAs) enhance glucose-dependent insulin secretion, suppress glucagon secretion, delay gastric emptying, and promote satiety (33, 34). Beyond glycemic control, GLP-1RAs reduce body weight and visceral adiposity and may modulate inflammation, oxidative stress, and other pathways implicated in cardiovascular and kidney injury (33, 35). These mechanisms may contribute to cardiorenal effects beyond glucose lowering.

Cardiovascular outcomes: In LEADER, liraglutide reduced major adverse cardiovascular events (MACE) by 13% (HR 0.87; 95% CI 0.78–0.97; ARR 1.9%; NNT 53 over 3.8 years) (11). In SUSTAIN-6, semaglutide was noninferior to placebo for MACE, with a 26% lower relative risk (HR 0.74; 95% CI 0.58–0.95; P<0.001 for noninferiority); nominal superiority was observed (P = 0.02), although superiority testing was not prespecified. Nonfatal stroke was reduced by 39% (HR 0.61; 95% CI 0.38–0.99) (36). REWIND demonstrated a 12% reduction in MACE with dulaglutide (HR 0.88; 95% CI 0.79–0.99) in a broader population that included many participants without established CVD (37). SELECT extended these findings to adults with overweight or obesity and established CVD but without diabetes, in whom semaglutide 2.4 mg reduced MACE by 20% (HR 0.80; 95% CI 0.72–0.90; ARR 1.5%; NNT 67 over approximately 3.3 years) (38). The benefit observed in SELECT despite the absence of diabetes suggests that the cardiovascular effects of semaglutide are not explained solely by glucose lowering.

Cardiovascular benefit has not been uniform across the class. EXSCEL with exenatide and ELIXA with lixisenatide established cardiovascular safety but did not show superiority for MACE (39, 40). Differences among individual agents, study populations, treatment exposure, and trial design may contribute to the variation in cardiovascular outcomes.

Kidney outcomes: FLOW enrolled 3,533 participants with T2D and CKD and found a 24% lower risk of the primary kidney composite outcome with semaglutide 1.0 mg compared with placebo (HR 0.76; 95% CI 0.66–0.88; ARR 3.8%; NNT 26 over a median of 3.4 years). MACE was also reduced by 18% (HR 0.82; 95% CI 0.68–0.98) (41). FLOW provides direct randomized outcome evidence for kidney protection with semaglutide in patients with T2D and CKD.

Heart failure outcomes: In STEP-HFpEF, semaglutide 2.4 mg improved health status assessed by the Kansas City Cardiomyopathy Questionnaire, exercise capacity, body weight, and C-reactive protein levels in patients with obesity-related HFpEF (42). Evidence in HFrEF remains less certain, with earlier studies reporting neutral or potentially unfavorable results (42).

Across randomized GLP-1RA trials, a recent meta-analysis reported lower risks of MACE (RR 0.86; 95% CI 0.80–0.93) and kidney composite outcomes (RR 0.79; 95% CI 0.73–0.86), with broadly consistent effects across several clinically relevant subgroups (43). The magnitude and nature of benefit, however, may vary according to the individual agent, clinical population, and underlying cardiovascular and kidney phenotype.

Safety and implementation considerations. Gastrointestinal adverse effects, particularly nausea, vomiting, and diarrhea, are the most common tolerability concerns and may lead to treatment discontinuation. Gallbladder and biliary events are also recognized, whereas pancreatitis is uncommon (44). Cost, availability, and access may further limit clinical uptake, particularly in low- and middle-income countries.

#### 3.1.2 Dual incretin therapies

Tirzepatide, a dual glucose-dependent insulinotropic polypeptide (GIP) and GLP-1 receptor agonist, produces substantial weight loss and improves glycemic control through complementary incretin pathways (45). In SURMOUNT-1, mean weight loss reached 20.9% with tirzepatide 15 mg (46). SUMMIT extended the evidence to obesity-related HFpEF, where tirzepatide reduced the risk of worsening HF events or cardiovascular death by 38% compared with placebo (HR 0.62; 95% CI 0.41–0.95) (45). Cardiovascular outcome data further inform its use in patients with T2D and established atherosclerotic CVD (**Table 1**) (47). Evidence for kidney outcomes remains limited, and whether the greater weight loss achieved with tirzepatide translates into greater cardiovascular or kidney protection than other incretin-based therapies remains uncertain.

**Table 1:** Major clinical outcome evidence for metabolic and adiposity-targeted therapies relevant to CKM health.

Therapy	Study/population	Primary outcome	Effect
Liraglutide	LEADER (11); T2D with high CV risk	CV death, nonfatal MI, or nonfatal stroke	HR 0.87 (95% CI 0.78–0.97)
Semaglutide	SUSTAIN-6 (36); T2D with high CV risk	CV death, nonfatal MI, or nonfatal stroke	HR 0.74 (95% CI 0.58–0.95)
Dulaglutide	REWIND (37); T2D with CV risk factors or established CVD	CV death, nonfatal MI, or nonfatal stroke	HR 0.88 (95% CI 0.79–0.99)
Semaglutide	SELECT (38); overweight/obesity and established CVD without diabetes	CV death, nonfatal MI, or nonfatal stroke	HR 0.80 (95% CI 0.72–0.90)
Semaglutide	FLOW (41); T2D and CKD	Major kidney events, ≥50% sustained eGFR decline, or kidney/CV death	HR 0.76 (95% CI 0.66–0.88)
Semaglutide	STEP-HFpEF (41); obesity-related HFpEF	Change in KCCQ-CSS and body weight	+16.6 points and −13.3%, respectively
Tirzepatide	SURMOUNT-1 (46); obesity/overweight without diabetes	Percentage change in body weight and ≥5% weight reduction	Up to −20.9% mean body weight
Tirzepatide	SUMMIT (45); obesity and HFpEF	CV death or worsening HF event	HR 0.62 (95% CI 0.41–0.95)

CKD, chronic kidney disease; CKM, cardiovascular-kidney-metabolic; CV, cardiovascular; CVD, cardiovascular disease; eGFR, estimated glomerular filtration rate; HF, heart failure; HFpEF, heart failure with preserved ejection fraction; HR, hazard ratio; KCCQ-CSS, Kansas City Cardiomyopathy Questionnaire Clinical Summary Score; MI, myocardial infarction; T2D, type 2 diabetes. *Note:* The studies were conducted in populations with cardiovascular, kidney, or metabolic conditions relevant to CKM health rather than in cohorts defined specifically by CKM syndrome.

#### 3.1.3 Metabolic and bariatric surgery

Metabolic and bariatric surgery (MBS) produces substantial and sustained weight loss through metabolic and physiological changes involving incretin signaling, bile acid metabolism, gut microbial composition, and neurohormonal pathway (48). Long-term observational data from the Swedish Obese Subjects study associate MBS with lower cardiovascular morbidity and mortality compared with usual care (49). Reduced albuminuria and slower CKD progression have also been reported in patients with obesity and T2D (50). Roux-en-Y gastric bypass (RYGB) can achieve substantial weight loss and metabolic improvement but requires consideration of perioperative risk and long-term nutritional deficiencies, particularly iron, vitamin B12, folate, calcium, and vitamin D deficiencies (51). MBS may also be cost-effective in appropriately selected patients with obesity and related comorbidities (52).

### 3.2 SGLT2 inhibitors: targeting cardiorenal pathways

Sodium-glucose cotransporter 2 (SGLT2) inhibitors reduce proximal tubular glucose and sodium reabsorption, promoting glucosuria and natriuresis (53). Increased sodium chloride delivery to the macula densa modulates tubuloglomerular feedback, reducing intraglomerular pressure and hyperfiltration (53–55). Natriuresis and osmotic diuresis may reduce congestion and cardiac filling pressures (54, 56), while other proposed mechanisms include favorable changes in myocardial energetics (52, 57), attenuation of inflammatory and fibrotic signaling, and modest reductions in blood pressure and arterial load (53, 54).

Kidney outcomes: In CREDENCE, canagliflozin reduced the primary kidney and cardiovascular composite outcome by 30% among patients with T2D and CKD (HR 0.70; 95% CI 0.59–0.82) (58). DAPA-CKD broadened this evidence to CKD with and without diabetes, with dapagliflozin reducing the primary composite outcome by 39% (HR 0.61; 95% CI 0.51–0.72) (59). EMPA-KIDNEY included a broader CKD population, including patients with an eGFR as low as 20 mL/min/1.73 m², and reported a 28% lower risk of kidney disease progression or cardiovascular death with empagliflozin (HR 0.72; 95% CI 0.64–0.82) (60).

Heart failure outcomes: DAPA-HF and EMPEROR-Reduced reported lower risks of worsening HF events and cardiovascular outcomes with SGLT2 inhibition in HFrEF (61, 62). EMPEROR-Preserved and DELIVER extended the clinical benefit to patients with mildly reduced or preserved ejection fraction (10, 63). Meta-analytic evidence similarly reports reductions in HF hospitalization across clinically relevant subgroups (64).

Cardiovascular outcomes: In EMPA-REG OUTCOME, empagliflozin reduced cardiovascular mortality by 38% (HR 0.62; 95% CI 0.49–0.77) (10). The CANVAS Program reported a 14% reduction in MACE with canagliflozin (HR 0.86; 95% CI 0.75–0.97), alongside an increased risk of lower-limb amputation that has not been consistently reproduced in subsequent trials (65). In DECLARE-TIMI 58, dapagliflozin met noninferiority criteria for MACE (HR 0.93; 95% CI 0.84–1.03; P<0.001 for noninferiority) and reduced cardiovascular death or HF hospitalization (HR 0.83; 95% CI 0.73–0.95), largely through fewer HF hospitalizations (HR 0.73; 95% CI 0.61–0.88) (66).

The cardiovascular and kidney effects of SGLT2 inhibitors have been observed across diverse clinical populations, although indications and evidence for specific outcomes vary among individual agents (21). Important adverse effects include genital mycotic infections, volume depletion, and uncommon diabetic ketoacidosis. Temporary interruption is recommended during clinical circumstances associated with increased ketoacidosis risk, including major surgery or prolonged fasting (67).

### 3.3 Targeting RAAS and mineralocorticoid receptor signaling

Renin-angiotensin-aldosterone system (RAAS) activation contributes to glomerular hypertension, sodium retention, inflammation, and fibrosis, linking cardiovascular and kidney injury within the CKM spectrum. Pharmacologic interruption of this pathway provides organ protection beyond blood pressure lowering.

#### 3.3.1 ACE inhibitors and ARBs

Angiotensin-converting enzyme inhibitors (ACEis) and angiotensin receptor blockers (ARBs) reduce angiotensin II-mediated glomerular hypertension, albuminuria, and progressive kidney injury. RENAAL and IDNT established the kidney-protective role of ARBs in diabetic kidney disease, with effects extending beyond blood pressure reduction alone (68, 69). RAAS inhibition is also foundational in HFrEF and selected populations at high cardiovascular risk (70, 71). Potential adverse effects include hyperkalemia, hypotension, and acute reductions in kidney function, while ACEi-associated cough may require substitution with an ARB (72). Dual RAAS blockade is generally avoided because of increased adverse events without sufficient additional clinical benefit (73, 74).

#### 3.3.2 Nonsteroidal mineralocorticoid receptor antagonists

Finerenone is a selective, nonsteroidal mineralocorticoid receptor (MR) antagonist with pharmacologic properties distinct from steroidal MR antagonists. Its lack of androgen and progesterone receptor activity reduces sex hormone-related adverse effects associated with spironolactone (75, 76).

In FIDELIO-DKD, finerenone reduced the primary kidney composite outcome by 18% (HR 0.82; 95% CI 0.73–0.93) and the key secondary cardiovascular composite outcome by 14% (HR 0.86; 95% CI 0.75–0.99) among patients with T2D and CKD receiving optimized RAAS inhibition (75). FIGARO-DKD reported a 13% reduction in the primary cardiovascular composite outcome (HR 0.87; 95% CI 0.76–0.98), largely attributable to fewer HF hospitalizations (HR 0.71; 95% CI 0.56–0.90) (76). In the prespecified FIDELITY pooled analysis of 13,026 participants, finerenone was associated with lower risks of cardiovascular (HR 0.86; 95% CI 0.78–0.95) and kidney composite outcomes across a broad spectrum of CKD associated with T2D (77) (**Table 2**).

**Table 2:** Major trials of cardiorenal therapies relevant to CKM health.

Therapy	Key trial/population	Primary outcome	Effect
Canagliflozin	CREDENCE (58); T2D and CKD	ESKD, doubling of serum creatinine, or kidney/CV death	HR 0.70 (95% CI 0.59–0.82)
Dapagliflozin	DAPA-CKD (59); CKD with or without T2D	≥50% sustained eGFR decline, ESKD, or kidney/CV death	HR 0.61 (95% CI 0.51–0.72)
Empagliflozin	EMPA-KIDNEY (60); CKD with or without diabetes	Kidney disease progression or CV death	HR 0.72 (95% CI 0.64–0.82)
Dapagliflozin	DAPA-HF (61); HFrEF with or without T2D	Worsening HF or CV death	HR 0.74 (95% CI 0.65–0.85)
Empagliflozin	EMPEROR-Reduced (62); HFrEF with or without T2D	HF hospitalization or CV death	HR 0.75 (95% CI 0.65–0.86)
Empagliflozin	EMPEROR-Preserved (10); HFpEF	HF hospitalization or CV death	HR 0.79 (95% CI 0.69–0.90)
Dapagliflozin	DELIVER (63); HFmrEF/HFpEF	Worsening HF or CV death	HR 0.82 (95% CI 0.73–0.92)
Finerenone	FIDELIO-DKD (75); T2D and CKD	Kidney failure, ≥40% sustained eGFR decline, or kidney death	HR 0.82 (95% CI 0.73–0.93)
Finerenone	FIGARO-DKD (76); T2D and CKD	CV death, nonfatal MI, nonfatal stroke, or HF hospitalization	HR 0.87 (95% CI 0.76–0.98)
Finerenone	FIDELITY (77); pooled T2D and CKD population	CV death, nonfatal MI, nonfatal stroke, or HF hospitalization	HR 0.86 (95% CI 0.78–0.95)

CKD, chronic kidney disease; CKM, cardiovascular-kidney-metabolic; CV, cardiovascular; eGFR, estimated glomerular filtration rate; ESKD, end-stage kidney disease; HF, heart failure; HFmrEF, heart failure with mildly reduced ejection fraction; HFpEF, heart failure with preserved ejection fraction; HFrEF, heart failure with reduced ejection fraction; HR, hazard ratio; MI, myocardial infarction; T2D, type 2 diabetes. *Note:* The trials were conducted in populations with cardiovascular, kidney, or metabolic conditions relevant to CKM health rather than in cohorts defined specifically by CKM syndrome.

Whether finerenone provides similar clinical benefits in CKD without diabetes remains uncertain, and evidence in this population is still evolving (78). Steroidal MR antagonists, including spironolactone and eplerenone, reduce morbidity and mortality in HFrEF (79, 80), although hyperkalemia and worsening kidney function can limit their use in CKD. Serum potassium and kidney function should therefore be monitored at treatment initiation and during follow-up.

### 3.4 Targeting lipid and atherosclerotic pathways

Atherogenic lipoproteins remain major contributors to cardiovascular risk across the CKM spectrum. Statins inhibit 3-hydroxy-3-methylglutaryl-coenzyme A (HMG-CoA) reductase, reducing hepatic cholesterol synthesis and increasing LDL receptor expression. Large-scale meta-analyses report an approximately 20% reduction in major vascular events per 1 mmol/L reduction in LDL-C across a broad range of cardiovascular risk (81, 82). Statins therefore remain foundational for ASCVD prevention in patients with appropriate indications. In SHARP, simvastatin plus ezetimibe reduced major atherosclerotic events by 17% among 9,270 patients with CKD (83). Statin-associated muscle symptoms are an important tolerability consideration, and statin therapy is associated with a modest increase in incident diabetes, although cardiovascular benefits generally outweigh this risk in appropriately selected patients (84, 85).

Ezetimibe reduces intestinal cholesterol absorption and provides additional LDL-C lowering when combined with statin therapy. In IMPROVE-IT, simvastatin plus ezetimibe reduced cardiovascular events compared with simvastatin alone after acute coronary syndrome (HR 0.936; 95% CI 0.89–0.99) (86).

Proprotein convertase subtilisin/kexin type 9 (PCSK9) inhibitors provide substantial additional LDL-C lowering. Evolocumab reduced the primary cardiovascular composite outcome by 15% in FOURIER (HR 0.85; 95% CI 0.79–0.92) (87), while alirocumab produced a similar reduction after acute coronary syndrome in ODYSSEY OUTCOMES (HR 0.85; 95% CI 0.78–0.93) (88). Cost and access may limit their use, particularly in low- and middle-income countries.

Bempedoic acid inhibits ATP-citrate lyase upstream of HMG-CoA reductase. In CLEAR Outcomes, involving 13,970 statin-intolerant patients, bempedoic acid reduced MACE by 13% (HR 0.87; 95% CI 0.79–0.96), providing an alternative for patients unable to tolerate recommended statin therapy (89) (**Table 3**).

**Table 3:** Major trials of lipid and atherosclerotic therapies relevant to CKM health.

Therapy	Key trial/population	Primary outcome	Effect
Simvastatin and ezetimibe	SHARP (83); CKD	Major atherosclerotic events	17% relative risk reduction
Simvastatin and ezetimibe	IMPROVE-IT (86); recent ACS	CV death, major coronary event, or nonfatal stroke	HR 0.94 (95% CI 0.89–0.99)
Evolocumab	FOURIER (87); established ASCVD receiving statin therapy	CV death, MI, stroke, hospitalization for unstable angina, or coronary revascularization	HR 0.85 (95% CI 0.79–0.92)
Alirocumab	ODYSSEY OUTCOMES (88); recent ACS receiving intensive statin therapy	CHD death, nonfatal MI, ischemic stroke, or hospitalization for unstable angina	HR 0.85 (95% CI 0.78–0.93)
Bempedoic acid	CLEAR Outcomes (89); statin-intolerant patients with or at high risk for CVD	CV death, nonfatal MI, nonfatal stroke, or coronary revascularization	HR 0.87 (95% CI 0.79–0.96)

ACS, acute coronary syndrome; ASCVD, atherosclerotic cardiovascular disease; CHD, coronary heart disease; CKD, chronic kidney disease; CKM, cardiovascular-kidney-metabolic; CV, cardiovascular; CVD, cardiovascular disease; HR, hazard ratio; MI, myocardial infarction. *Note:* The trials were conducted in populations with cardiovascular, kidney, or metabolic conditions relevant to CKM health rather than in cohorts defined specifically by CKM syndrome.

Inclisiran, a small interfering RNA (siRNA) targeting hepatic PCSK9 messenger RNA, provides sustained LDL-C lowering with twice-yearly maintenance dosing. Whether this reduction translates into fewer cardiovascular events is being evaluated in cardiovascular outcome trials (90). Lipoprotein(a)-targeted therapies, including olpasiran, achieve substantial reductions in Lp(a), but their role in routine CKM management will depend on evidence from cardiovascular outcome trials (91).

### 3.5 Targeting heart failure pathways

Heart failure phenotypes differ in pathophysiology and therapeutic response, requiring distinct treatment strategies within the CKM spectrum (92). In HFrEF, systolic dysfunction is accompanied by neurohormonal activation and adverse ventricular remodeling. Guideline-directed medical therapy combines an angiotensin receptor-neprilysin inhibitor (ARNI), or an ACEi/ARB when appropriate, an evidence-based beta-blocker, a mineralocorticoid receptor antagonist, and an SGLT2 inhibitor (93). In PARADIGM-HF, sacubitril/valsartan reduced cardiovascular death or HF hospitalization compared with enalapril (HR 0.80; 95% CI 0.73–0.87) (94).

HFpEF is more heterogeneous, with obesity, metabolic dysfunction, systemic inflammation, microvascular dysfunction, and myocardial remodeling contributing to disease expression in many patients (90). SGLT2 inhibitors reduce worsening HF events across HFpEF populations, as reported in EMPEROR-Preserved and DELIVER (61, 62). Obesity-related HFpEF provides an additional opportunity for phenotype-directed treatment. Semaglutide improved symptoms, physical function, and body weight in STEP-HFpEF (95), while tirzepatide reduced worsening HF events or cardiovascular death in SUMMIT (45).

The role of GLP-1RAs in HFrEF remains uncertain. Earlier studies have not demonstrated clear HF-specific clinical benefit, and current evidence does not support their use specifically as HFrEF-directed therapy (42). However, GLP-1RAs may still be indicated for coexisting conditions such as T2D, obesity, or ASCVD. The differing evidence across HF phenotypes highlights the importance of matching therapeutic strategies to the dominant clinical phenotype and available outcome evidence.

## 4 Phenotype-directed CKM therapy

### 4.1 Rationale and distinction from CKM staging

Patients across the CKM spectrum share interconnected cardiovascular, kidney, and metabolic pathways, but the dominant clinical manifestations and sources of residual risk vary considerably. The phenotype-directed framework proposed in this review is intended to complement, rather than replace, the AHA CKM staging system. CKM stages describe disease progression and risk across the cardiovascular-kidney-metabolic continuum, whereas phenotyping identifies the predominant clinical features that may influence therapeutic priorities (7).

Four overlapping phenotypes are proposed: obesity-dominant CKM, characterized primarily by excess or dysfunctional adiposity and associated metabolic abnormalities; albuminuric CKD-dominant CKM, characterized by albuminuria and progressive kidney dysfunction; HF-dominant CKM, encompassing HFrEF and HFpEF; and ASCVD-dominant CKM, characterized by subclinical or established atherosclerotic disease. These phenotypes broadly span different CKM stages and are intended to facilitate therapeutic prioritization rather than define new disease categories (1).

Whether phenotype-based treatment improves clinical outcomes beyond established disease-specific and risk-based approaches remains uncertain. Considerable overlap is expected, particularly in advanced CKM, where obesity, CKD, HF, ASCVD, and metabolic abnormalities frequently coexist. Moreover, evidence informing phenotype-directed treatment derives predominantly from trials conducted in disease-specific populations rather than prospective RCTs stratified according to CKM phenotype. The proposed phenotypes should therefore be regarded as a pragmatic conceptual framework for organizing therapeutic priorities, not as validated or mutually exclusive treatment algorithms.

### 4.2 Identifying phenotypes in clinical practice

Phenotype assessment can be based largely on routinely available clinical, laboratory, and imaging information (7). Obesity-dominant CKM may be identified through measures of adiposity, including BMI and waist circumference, together with dysglycemia, dyslipidemia, or hypertension. Albuminuric CKD-dominant CKM is characterized primarily by reduced eGFR and/or elevated UACR, particularly persistent albuminuria or progressive loss of kidney function. HF-dominant CKM is identified from clinical features of HF supported by natriuretic peptides and cardiac imaging, with further classification according to left ventricular ejection fraction. ASCVD-dominant CKM includes established coronary, cerebrovascular, or peripheral artery disease and may also encompass appropriately identified subclinical atherosclerosis (**Table 4**).

**Table 4:** Proposed phenotype-directed therapeutic priorities across CKM stages.

Dominant phenotype	Typical CKM stage(s)	Therapeutic priorities	Key considerations
Obesity-dominant	1–2	Lifestyle intervention; GLP-1RA or dual incretin therapy when indicated; MBS in eligible patients	Address dysglycemia, hypertension, dyslipidemia, CKD, and HF according to established indications
Albumi nuric CKD-dominant	2–4	ACEi/ARB when indicated and tolerated; SGLT2i; finerenone in eligible patients with T2D and CKD	Monitor eGFR, potassium, blood pressure, and tolerability; consider GLP-1RA for appropriate metabolic or CV indications
HF-dominant: HFrEF	3–4	ARNI, or ACEi/ARB when appropriate; evidence-based beta-blocker; MRA; SGLT2i	Initiate and optimize GDMT as tolerated; use GLP-1RA according to coexisting indications rather than as HFrEF-directed therapy
HF-dominant: HFpEF	3–4	SGLT2i; diuretics for congestion; incretin-based therapy in eligible patients with obesity	Address obesity, CKD, T2D, hypertension, and other contributing conditions
ASCVD-dominant	3–4	High-intensity or maximally tolerated statin; antiplatelet therapy when indicated; additional LDL-C lowering according to residual risk	Consider ezetimibe, PCSK9 inhibition, or bempedoic acid according to LDL-C, risk, and statin tolerance
Multiple overlapping phenotypes	2–4	Layer therapies with established outcome benefits according to each clinical indication	Consider kidney function, potassium, blood pressure, tolerability, treatment burden, affordability, and access

ACEi, angiotensin-converting enzyme inhibitor; ARB, angiotensin receptor blocker; ARNI, angiotensin receptor-neprilysin inhibitor; ASCVD, atherosclerotic cardiovascular disease; CKD, chronic kidney disease; CKM, cardiovascular-kidney-metabolic; CV, cardiovascular; eGFR, estimated glomerular filtration rate; GDMT, guideline-directed medical therapy; GLP-1RA, glucagon-like peptide-1 receptor agonist; HF, heart failure; HFpEF, heart failure with preserved ejection fraction; HFrEF, heart failure with reduced ejection fraction; MBS, metabolic and bariatric surgery; MRA, mineralocorticoid receptor antagonist; PCSK9, proprotein convertase subtilisin/kexin type 9; SGLT2i, sodium-glucose cotransporter 2 inhibitor; T2D, type 2 diabetes; UACR, urinary albumin-to-creatinine ratio.

The phenotypes and therapeutic priorities shown are a conceptual framework proposed in this review and are not formal AHA CKM categories or validated treatment algorithms. Individual treatment should be guided by CKM stage, established clinical indications, comorbidities, kidney function, tolerability, and patient-specific factors.

Emerging approaches, including multiomics, advanced cardiovascular imaging, and artificial intelligence-based risk stratification, may enable more granular characterization of CKM heterogeneity (96, 97). Their role in defining clinically actionable CKM phenotypes, however, requires prospective validation.

### 4.3 Combination therapy: evidence, controversies, and limitations

Combination therapy is biologically attractive in CKM syndrome because SGLT2 inhibitors, GLP-1RAs, finerenone, and RAAS inhibitors act through complementary pathways. Mechanistic complementarity, however, does not establish clinical synergy, particularly when incremental effects on hard clinical outcomes remain uncertain.

The CONFIDENCE trial evaluated simultaneous initiation of finerenone and empagliflozin compared with either treatment alone in patients with T2D and CKD. Combination therapy produced a greater reduction in UACR than either monotherapy (98). The trial was not designed to determine incremental effects on kidney failure, MACE, HF hospitalization, or mortality, limiting conclusions about synergy for hard clinical outcomes.

Evidence for combined SGLT2 inhibitor and GLP-1RA therapy is also evolving. Analyses of randomized trials report cardiovascular and kidney benefits of each class irrespective of background use of the other therapy (99, 100). Concomitant treatment may therefore be appropriate when both therapies are clinically indicated, although superiority of the combination over either class alone for hard clinical outcomes has not been established. Observational studies have reported additional cardiorenal benefits, but residual confounding and treatment-selection bias limit causal interpretation.

Several multidrug strategies are already established for specific clinical indications, including RAAS inhibition with SGLT2 inhibition in CKD, addition of finerenone in eligible patients with T2D and CKD, multidrug guideline-directed therapy for HFrEF, and intensification of LDL-C lowering when required (77, 91, 101). These approaches should be distinguished from unproven pharmacological synergy.

Layering multiple therapies also increases the need to monitor kidney function, serum potassium, blood pressure, and volume status. Polypharmacy, adherence, treatment burden, affordability, and access should be considered when individualizing treatment, particularly in resource-constrained settings.

## 5 Emerging mechanism-based therapies

Despite substantial advances in CKM therapy, residual cardiovascular, kidney, and metabolic risk persists, creating opportunities for therapies directed at pathways not fully addressed by established treatments (102).

### 5.1 Anti-inflammatory strategies: colchicine

Colchicine inhibits microtubule polymerization and modulates inflammatory pathways, including neutrophil activation and NLRP3 inflammasome signaling (103). In LoDoCo2, colchicine reduced the primary cardiovascular composite outcome by 31% in patients with chronic coronary disease (HR 0.69; 95% CI 0.57–0.83; ARR 2.5%; NNT 40) (104). In COLCOT, conducted after myocardial infarction, the primary cardiovascular composite outcome was reduced by 23% (HR 0.77; 95% CI 0.61–0.96) (105). Its role beyond ASCVD remains uncertain because CKM-specific trial evidence is lacking. Gastrointestinal intolerance and increased toxicity risk in advanced kidney impairment or with interacting medications are important considerations.

### 5.2 Aldosterone synthase inhibitors: baxdrostat

Baxdrostat selectively inhibits aldosterone synthase (CYP11B2), reducing aldosterone production while aiming to preserve cortisol synthesis (106). Clinical trials have reported meaningful blood pressure reductions in resistant or uncontrolled hypertension (107). Cardiovascular and kidney outcome data remain limited, and whether aldosterone synthase inhibition provides additional benefit over established MR antagonism is uncertain. Longer-term evaluation is also needed to characterize hyperkalemia risk and potential effects on adrenal steroidogenesis (23).

### 5.3 Endothelin receptor antagonists

Endothelin signaling contributes to vasoconstriction, inflammation, fibrosis, and kidney injury. In SONAR, the selective endothelin A receptor antagonist atrasentan reduced the primary kidney composite outcome in patients with T2D and CKD (HR 0.65; 95% CI 0.49–0.88) (108). In ZENITH-CKD, zibotentan combined with dapagliflozin produced greater albuminuria reduction than dapagliflozin alone (108).

Fluid retention and the potential for HF remain important safety concerns. The enrichment design used in SONAR may also limit generalizability, while evidence for cardiovascular mortality, MACE, and HF outcomes remains limited. The clinical value of adding endothelin receptor antagonism to contemporary CKM therapy therefore requires further evaluation.

### 5.4 Next-generation incretin therapies

In SOUL, oral semaglutide reduced MACE by 14% in patients with T2D and ASCVD and/or CKD (HR 0.86; 95% CI 0.77–0.96) (109), extending cardiovascular outcome evidence for GLP-1RA therapy to an oral formulation. Orforglipron, a nonpeptide oral GLP-1 receptor agonist, has produced substantial weight loss and improvements in glycemic control (110), although cardiovascular and kidney outcome data remain limited.

Retatrutide, a GIP, GLP-1, and glucagon receptor agonist, produced substantial weight loss in a phase 2 trial among adults with obesity (111). Whether the greater weight loss achieved with newer incretin-based therapies translates into greater cardiovascular or kidney protection remains uncertain and requires clinical outcome evidence.

### 5.5 RNA-targeting lipid therapies

Inclisiran, a small interfering RNA (siRNA) targeting hepatic PCSK9 messenger RNA, produces sustained LDL-C reductions of approximately 50% with twice-yearly maintenance dosing (88, 89). Olpasiran, an siRNA targeting apolipoprotein(a) synthesis, produces substantial reductions in circulating lipoprotein(a) (112). Whether these lipid reductions translate into fewer cardiovascular events remains under evaluation in clinical outcome trials. Although elevated lipoprotein(a) is a causal risk factor for ASCVD, the magnitude of cardiovascular risk reduction achievable through pharmacologic lipoprotein(a) lowering has not yet been established (113).

## 6 Potential developments and future directions

Advancing CKM care will require research strategies that reflect the interconnected cardiovascular, kidney, and metabolic biology of the syndrome rather than treating each component in isolation. Key priorities include CKM-specific trial designs, optimization of combination therapy, improved risk stratification, equitable implementation, and greater emphasis on patient-centered outcomes.

### 6.1 CKM-specific clinical trials

Most therapeutic evidence originates from trials designed around individual conditions such as T2D, CKD, HF, obesity, or ASCVD rather than CKM syndrome as an integrated clinical entity. Trials incorporating CKM staging could determine whether treatment effects vary across stages and whether phenotype-directed strategies improve cardiovascular, kidney, metabolic, and patient-centered outcomes.

### 6.2 Optimizing combination therapy

As therapies with multiorgan benefits expand, important questions remain regarding treatment sequencing, combination, and potential interactions among SGLT2 inhibitors, GLP-1-based therapies, RAAS inhibitors, finerenone, and emerging agents. Factorial, adaptive, and pragmatic trial designs may help distinguish additive effects from true pharmacological synergy while accounting for safety, polypharmacy, treatment burden, and cost.

### 6.3 Precision risk stratification

Combining established clinical variables with biomarkers, genetics, multiomics, advanced imaging, and artificial intelligence may improve CKM risk stratification and treatment selection. Translation into clinical practice will require prospective validation, assessment of discrimination, calibration, and clinical utility, and evaluation across diverse populations and health care settings.

### 6.4 Implementation, access, and equity

The population-level impact of CKM therapies will depend on their effective translation into routine care. Research should examine multidisciplinary care models, implementation strategies, affordability, medication access, adherence, and health-system barriers, particularly in low- and middle-income countries. Pragmatic trials and health-economic evaluations will be important for determining how evidence-based CKM care can be delivered equitably and sustainably.

### 6.5 Patient-centered CKM care

Clinical trials and implementation studies should increasingly incorporate outcomes that matter directly to patients, including symptoms, functional status, quality of life, treatment burden, and treatment preferences. Integrating these measures with cardiovascular and kidney outcomes may facilitate more individualized CKM management and shared decision-making.

### 6.6 Limitations of the current evidence base

Many pivotal trials informing CKM therapy were industry sponsored. Although industry sponsorship does not itself indicate bias, its potential influence should be considered when interpreting the evidence, particularly for newer therapies with limited independent replication. Selective publication of positive findings also cannot be excluded. Because this narrative review did not formally assess risk of bias or publication bias, the extent to which these factors affect the available evidence cannot be determined. In addition, some emerging therapies are supported primarily by surrogate outcomes rather than cardiovascular or kidney events. Independent replication, transparent reporting of neutral and negative findings, and trials evaluating clinically meaningful outcomes would strengthen the evidence base for CKM therapy.

## 7 Conclusion

CKM syndrome provides an integrated framework for addressing the interconnected cardiovascular, kidney, and metabolic pathways that contribute to disease progression. Established therapies, including SGLT2 inhibitors, GLP-1-based therapies, RAAS inhibitors, mineralocorticoid receptor antagonists, and lipid-lowering agents, target complementary pathways and provide cardiovascular, kidney, or metabolic benefits across relevant patient populations. Emerging therapies targeting inflammation, aldosterone synthesis, endothelin signaling, incretin pathways, and lipid-related genetic targets may further expand therapeutic options, although their roles within integrated CKM care require additional clinical outcome evidence.

The phenotype-directed approach proposed in this review may complement CKM staging by helping prioritize therapy according to predominant clinical and mechanistic features, but prospective validation is required. Future progress will depend on CKM-specific trials, rigorous evaluation of combination strategies, improved risk stratification, and equitable implementation of evidence-based therapies across diverse health care settings.
